# Placental programming of metabolic diseases: molecular pathways from maternal pregnancy complications to offspring metabolic risk

**DOI:** 10.3389/fendo.2026.1860825

**Published:** 2026-10-26

**Authors:** Duo Zhou

**Affiliations:** Department of Medical Genetics and Prenatal Diagnosis, Hainan Women and Children’s Medical Center, Haikou, China

**Keywords:** fetal programming, gestational diabetes mellitus, hypertensive disorders of pregnancy, intergenerational impact, metabolic risk, placenta, placental dysfunction, placental programming

## Abstract

**Introduction:**

The placenta is increasingly recognized as a dynamic regulatory organ that translates both maternal and paternal physiological and pathological states into developmental signals into developmental signals shaping offspring health across the lifespan. In the context of maternal metabolic and obstetric complications, placental function extends far beyond the regulation of fetal growth, influencing key aspects of development including endocrine set points, nutrient allocation, inflammatory responses, vascular adaptation, and molecular programming across fetal organs.

**Results:**

This review examines how maternal conditions —such as obesity, gestational diabetes mellitus (GDM), hypertensive disorders of pregnancy, undernutrition, micronutrient imbalance, and maternal inflammatory stress —induce structural, endocrine, vascular, metabolic, and epigenetic alterations in the placenta. We further explore how these changes disrupt critical molecular pathways, including nutrient sensing and transport, oxidative stress responses, mitochondrial function, angiogenesis and glucocorticoid signaling, immune regulation, and gene expression. A central focus is the convergence of placental mechanisms across diverse maternal disorders, leading to shared offspring phenotypes such as insulin resistance, increased adiposity, dyslipidemia, impaired glucose regulation, altered appetite control, and elevated risk of cardiometabolic disease later in life.

**Conclusion:**

Additionally, we address sex-specific placental adaptations, fetal tissue vulnerability, and the translational potential of placental biomarkers and omics-based approaches supported by mechanistic experimental models. By positioning the placenta as the primary biological interface linking maternal complications to offspring susceptibility to endocrine and complex metabolic diseases, this review provides an integrated framework for understanding developmental programming in metabolic health.

## Introduction

The placenta holds an exceptionally unique position in developmental biology. It is the first organ to arise and serve embryonic life (1). In addition, it is the primary interface by which the maternal condition is converted into fetal developmental signal (2). Rather than simply functioning as a passive conduit for maternal nutrient supply and oxygen delivery, the placenta has developed multiple specialized function (sensory, transport, endocrine, immune and vascular) that continually interpret the maternal environment and adjust fetal exposure to environmental influences (3). This process is key to the developmental origin of disease paradigm. This paradigm asserts that chronic disease risk may be biologically embedded at a time when there are “windows” of prenatal developmental sensitivity (4). Therefore, metabolic disease occurring in adulthood, previously viewed solely as a result of adult lifestyle choices and genetic inheritance, will also occur due to prenatal developmental programming events that have altered fetal organ structure, endocrine responsiveness, metabolic flexibility and homeostatic set points prior to birth.

The early interest in prenatal programming came out of epidemiological studies which showed that low birth weights associated with restricted fetal development were correlated with increased risks of developing type 2 diabetes, central obesity, high blood pressure (hypertension) and heart disease later in life (5). Those findings shifted focus away from the postnatal environmental exposures to the intrauterine (fetal/embryonic/maternal) environment as another important biologically shaping factor (6). Later on, this concept was extended beyond just nutritional deprivation during fetal development to include maternal obesity, gestational diabetes mellitus, hypertensive disorders of pregnancy, maternal systemic inflammation, maternal psychosocial stress, endocrine disturbances and micro-nutritional deficiencies.

While maternal contributions to the intrauterine environment are well-established, emerging evidence highlights the significance of paternal metabolic health as a preconception determinant of placental function. Paternal obesity, metabolic syndrome, and dietary exposures prior to conception can induce epigenetic remodeling in spermatozoa—including alterations in DNA methylation, histone modifications (e.g., H3K4me3), and small non-coding RNAs. These epigenetic signatures are transmitted at fertilization and have been shown to map onto placental chromatin and transcriptomic profiles, thereby influencing placental vascularization, nutrient transport efficiency, and stress responses independent of the maternal environment.

The placenta, which develops during normal physiological development, is responsible for coordinating fetal growth with precision (7, 8). The placenta controls the exchange of glucose, amino acids, fatty acids, micronutrients and oxygen (9). It also creates hormones for maternal adaptation during pregnancy, controls maternal-fetal immune tolerance and maintains the appropriate vascular structure required for gestation (10, 11). However, maintaining this equilibrium can be disrupted by a variety of maternal metabolic and obstetric complications. For example, maternal obesity and gestational diabetes can lead to the accumulation of lipids within the placenta, cause inflammation, create oxidative stress and disrupt nutrient sensing (12, 13). These conditions expose the fetus to excessive nutrients and endocrine disrupting substances.

Placental programming is significant because it indicates that fetal development relies not only on the delivery of nutrients, but on how those nutrients are signaled by rapidly developing organs. Organ systems undergo rapid differentiation throughout gestation (14, 15). As such, pancreatic beta cell development, hypothalamus development of appetite circuits, adipocyte lineage commitment, skeletal muscle metabolic capabilities, and hepatic glucose lipid regulation, are all extremely pliable (16, 17). A disruption in placental functioning during the time when these organ systems are rapidly developing will result in a change in either the timing of, or the degree to which the fetus experiences various types of hormones, inflammation and nutrient exposure. These alterations can continue well beyond the initial prenatal stimulus for them. Programming may be present in the form of decreased insulin sensitivity, changed body composition, hypertriglyceridemia, hypertension, dysregulated appetite, accelerated “catch up” growth, or increased susceptibility to metabolic syndrome (18, 19). It is important to recognize that this does not occur through a single linear path way. Rather they represent the result of the interaction of many different placental functions, including; transporter expression abnormalities, dysfunctional mitochondria, ER stress, inflammatory responses, abnormal glucocorticoid metabolism, maladaptive endothelial response and epigenetic modification.

The role that the placenta plays in intergenerational metabolic diseases is strengthened when it comes to epigenetic regulation (20). The placenta is an extremely plastic organ with very high rates of cellular turnover and exposure to external environment; these characteristics make it an excellent model for studying how DNA methylation, histone modification, chromatin accessibility and non-coding RNA networks are affected by the maternal metabolic state (21). Therefore, because placental epigenetic modifications have the ability to act as both intermediate mechanisms in the transmission of transitory maternal perturbation and also potentially as biomarkers of risk for long-term phenotypic outcomes in offspring, they are of great interest (22).

A further significant trend is the increasing recognition that there are sexually distinct forms of placental response (23). While male and female placentas can react differently to maternal metabolic stress, maternal hypoxia, maternal inflammation, or maternal glucocorticoids, this difference in reaction could potentially explain some differences in how offspring grow prenatally and their subsequent postnatal metabolic health (24). This has wider implications; it illustrates that placental programming is not uniform, and therefore offspring susceptibility cannot be predicted based solely on maternal diagnosis. The same maternal complication will have different prenatal developmental effects as a function of the type of placenta an individual fetus has developed, the ability of the placenta to compensate for the maternal complication, and the specific intrauterine environment that an individual develops within. Therefore, placental biology represents a finer scale of analysis than isolated consideration of maternal diseases.

## Search methodology

### Literature search strategy

This review employed a structured, PRISMA-informed narrative synthesis to elucidate the mechanistic pathways linking maternal complications, placental dysfunction, and offspring metabolic risk. A comprehensive literature search was conducted across PubMed/MEDLINE, Scopus, Web of Science, the Cochrane Library, and ClinicalTrials.gov up to March 2026. The strategy combined controlled vocabulary (MeSH) and free-text terms using Boolean logic (AND/OR) to integrate three key domains: placental biology (“placenta,” “placental programming,” “trophoblast”), maternal exposures (“gestational diabetes,” “preeclampsia,” “maternal obesity,” “undernutrition”), and offspring outcomes (“insulin resistance,” “cardio-metabolic risk,” “epigenetics”). Reference lists of retrieved articles and Google Scholar were manually screened to identify additional mechanistic studies missed by electronic searches.

### Screening and selection process

Screening was performed independently by two reviewers using Covidence software, with discrepancies resolved through consensus or adjudication by a third senior reviewer. The process strictly followed the PRISMA 2020 flowchart, see **Figure 1** for details. Of 1,324 records identified (1,246 from databases, 78 from registers), 412 duplicates were removed. Subsequently, 912 records underwent title and abstract screening, of which 720 were excluded based on irrelevance to the mechanistic focus. The remaining 192 full-text articles were assessed for eligibility; 7 could not be retrieved, leaving 185 for full evaluation. Ultimately, 105 studies were included in the final synthesis. Exclusion reasons for the 80 rejected studies included insufficient mechanistic detail (*n* = 62), duplicate reporting or review articles (*n* = 12), and low methodological quality or inadequate sample sizes (*n* = 6).

**Figure 1:**
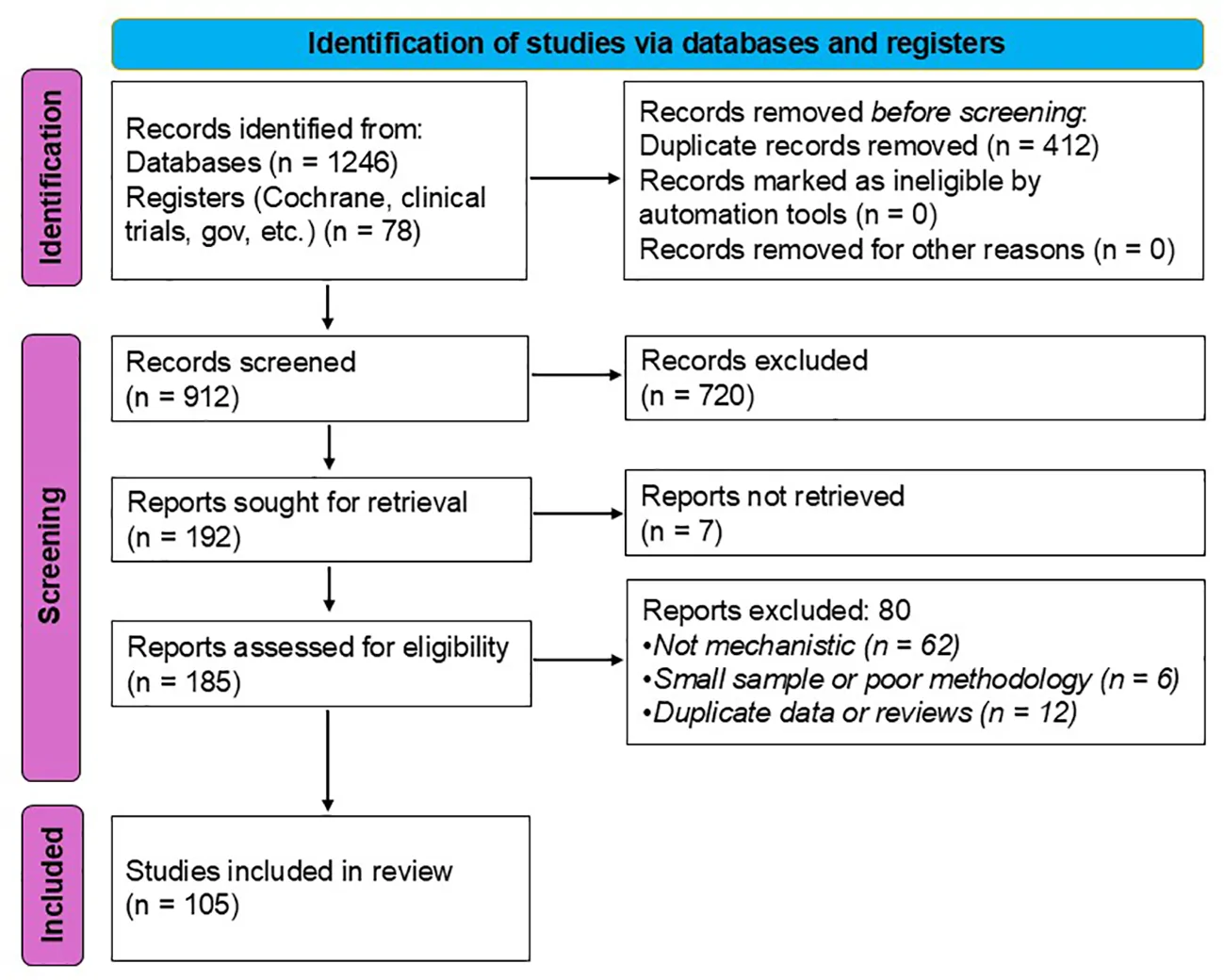
PRISMA flow diagram.

### Evidence synthesis and critical appraisal

Evidence was synthesized narratively with a critical appraisal of study quality and bias. We stratified the analysis by study design to address inherent limitations: human cohort studies were prioritized but noted for potential biases from cross-sectional placental sampling; animal models were assessed for species-specific differences and diet composition relevance; and *in vitro* studies were evaluated for physiological relevance. We explicitly acknowledge translational gaps, particularly that most epigenetic data derive from rodents, which differ significantly from human placental biology in structure and endocrine function. Furthermore, we highlight conflicting evidence regarding placental nutrient transporter expression in obesity versus GDM, likely attributable to varying gestational ages at sampling and diagnostic criteria. Studies were included if they reported a maternal complication, placental alterations, and fetal/offspring metabolic implications, excluding those focusing solely on immediate obstetric outcomes without developmental relevance. More exclusion and inclusion criteria are shown in **Table 1**.

**Table 1:** Inclusion and exclusion criteria.

Inclusion criteria	Exclusion criteria
Human, animal, and translational studies examining placental biology in relation to maternal complications	Studies unrelated to placental structure, function, or molecular signaling
Studies on maternal obesity, gestational diabetes mellitus, hypertensive disorders of pregnancy, undernutrition, micronutrient imbalance, inflammation, or endocrine stress	Studies focused only on immediate obstetric outcomes without mechanistic or endocrine-metabolic interpretation
Studies reporting placental molecular pathways such as inflammation, oxidative stress, mitochondrial dysfunction, angiogenesis, nutrient transport, endocrine signaling, or epigenetic regulation	Purely descriptive neonatal studies without placental relevance or developmental significance
Studies linking placental alterations to fetal programming or offspring endocrine-metabolic outcomes	Duplicate publications, reviews used as source material only, conference abstracts, and low-information commentaries
Original studies with sufficient methodological clarity and thematic relevance	Studies with very small samples, weak methodology, or inadequate mechanistic content

## The Placenta as the central interface in developmental programming

### Placental structure, functional zonation, and maternal-fetal exchange

The placenta is an extremely specialized temporary body part with separate compartments, structurally and functionally (25, 26). These provide an optimal exchange environment for both maternal and fetal bodies while maintaining their physiological segregation. Chorionic villi comprise the major exchange structures of the human placental unit; these project into the intervillous space bathing in maternal blood (27–29). Syncytiotrophoblasts provide the main site for material transfer, signal transmission and barrier properties (30). Cytotrophoblasts located beneath this layer replenish and differentiate into the trophoblastic population (31).

Capillaries containing fetal blood vessels are present within the central stroma of each chorionic villus along with stromal cells, macrophage cells and other non-vascular elements providing structural support to facilitate vascular exchange processes and local regulatory mechanisms (32). A subpopulation of extravillous trophoblasts extends from the villi into the decidua and maternal spiral arteries and through these actions remodel the uterine vasculature to establish a low-resistance, high-capacitance circulation facilitating growth of the fetus (33, 34). Through such compartmentalization of structure and function, the placenta operates not as a single tissue entity but rather as a functionally zoned organ whose various transport functions, hormone-producing capacities, vascular control mechanisms, and immunological responses are spatially organized.

Beginning with the need for tightly controlled mechanisms of transport (in addition to zonation) for maternal-fetal exchange and as opposed to purely diffusive exchanges, glucose is moved primarily by facilitative transporters, amino acids through active transporter systems, and fatty acid movement via protein-lipase-transporter combinations (35). As for oxygen delivery, it depends upon maternal perfusion, villous architecture, diffusion distance, and fetal blood flow (9). Micronutrients are transferred via selectively expressed carrier systems based upon developmental priorities (36). Thus, the placenta establishes both the amount and proportion of available nutrients to the developing fetus (2). In turn, changes in villous branching, trophoblastic surface area, barrier thickness, transporter number, mitochondrial density, and/or local blood flow can result in altered fetal exposures despite apparently equivalent amounts of each respective nutrient being present in maternal circulation.

### Endocrine, immune, and vascular roles of the placenta

Beyond exchange functions of placenta, placental function as a major endocrine organ shapes maternal physiology while also influencing fetal development through hormonal signaling (37). It produces a broad range of hormones, growth factors, cytokines, and bioactive peptides including human chorionic gonadotropin, human placental lactogen, progesterone, estrogens, leptin, corticotropin-releasing hormone and insulin-like growth factor-related mediators (38, 39). These signals assist to regulate the maternal state of insulin sensitivity, appetite, lipoid metabolism, vascular adaptation, and substrate mobilization during pregnancy, thus indirectly affecting fetal nutrient exposure. At the same time, placental endocrine activity contributes directly to the developmental environment experienced by the fetus, influencing growth trajectories, tissue differentiation and stress responsiveness. Placental glucocorticoid handling is especially important for this regard, as enzymes like 11β-hydroxysteroid dehydrogenase type 2 help to regulate fetal exposure to glucocorticoids from the mother (40, 41). Reduced protective metabolism of glucocorticoids can increase fetal exposure to stress hormones and can contribute to long term changes in insulin sensitivity, adiposity, blood pressure control and hypothalamic-pituitary-adrenal axis function (42).

The placenta is also an immunologically active organ, where it regulates a non-reactive state (tolerance) between the maternal and fetal tissues by having interaction with maternal immune cells, macrophages, natural killer cells, cytokines, chemokines, and extracellular vesicles (43, 44). In the case of inflammation in the maternal environment, the placenta responds appropriately (45, 46). It achieves this by maintaining tight regulation in the way that the above-mentioned components interact with each other. As long as there is no disturbance to this regulation under normal circumstances, then the placenta has regulatory mechanisms for promoting implantation, vascular remodelling, tissue turnover, and antimicrobial defences without activating harmful inflammatory processes. Any disturbances to these regulatory mechanisms could result in significant effects on development.

### Placental plasticity and developmental adaptation to maternal signals

The placenta has an enormous ability to adapt as pregnancy progresses (47). The placenta has this ability because it does not have the limitations on lineage and structure like most other fetal organ systems (48). So, the placenta can be changed as needed with respect to transporters, trophoblast differentiation, hormone production, mitochondria, blood vessel formation, oxidative damage prevention, inflammation mediation, and cell proliferation based on changes in maternal nutrition levels, oxygen delivery, hormones and inflammatory states (49). Therefore, the placenta’s ability to adapt is critical to supporting fetal development when maternal nutritional levels change and also contributes to variability in outcomes associated with maternal stress during pregnancy (50, 51). However, the ability of the placenta to adapt is limited and while some adaptations support fetal survival or growth at a given point in time they may direct fetal developmental pathways toward characteristics of a fetus that would be best suited for expected future stresses or nutrient deficiencies (52). Additionally, if the postnatal environment (e.g., postpartum) is different than what was predicted by the prenatally adapted placenta then the resulting mismatch could increase an individual’s risk of developing diseases later in life.

## Maternal complications that initiate placental programming

Placental “programming” can be a result of many different types of exposures (pathological) to maternal conditions and the resultant alterations in metabolism, hormones, vascularization, inflammation, oxidation and overall intrauterine environment (53). Maternal complications such as hypertension, pre-eclampsia, diabetes (gestational), nutritional deficiencies and others vary in their clinical manifestations but are likely to present with similar alterations in placental function related to nutrient uptake, endocrine signaling, vascular development, immunoregulation, mitochondrial function and redox status (54, 55). At the placental level, maternal complications produce structural adaptations such as changes in transporters, trophoblastic differentiation, inflammatory signaling and hormone production (56). Initially these responses are adaptive attempts to compensate for an adverse intrauterine condition to promote continued fetal growth or maintenance of placental efficiency. As a consequence of chronic, severe or inappropriate timing of maternal stress, however, what was once adaptive becomes maladaptive and results in the alteration of the biochemical signals that govern the development of fetal tissues (57). Therefore, maternal complications pose more than just short-term risks during pregnancy. They provide the molecular and physiological contexts in which the offspring’s long-term endocrine-metabolic susceptibility is established.

### Maternal obesity and excess nutrient exposure

Maternal obesity creates a metabolically dysfunctional intrauterine environment by increasing nutrient availability through excess calorie intake and creating chronic low-grade inflammation (58, 59). Thus, the placenta is not only exposed to an increased amount of substrate it is also subjected to hypermetabolic conditions that include elevated free fatty acids, altered adipokine profiles, hyperinsulinemia, oxidative stress and cytokines (46, 60). All these factors influence trophoblast behaviour, lipid handling, transporter expression and mitochondrial function. Tissue samples from placentae obtained at delivery from obese pregnancies frequently show evidence of increased lipid storage, enhanced inflammatory activation, altered glucose and amino acid uptakes, decreased mitochondrial efficiency and endoplasmic reticulum stress (55, 61). Therefore, the placenta in obese pregnancy represents a metabolically stressed organ which has to process excessive energy signals while maintaining its functions of exchange and hormone secretion (62, 63). Such condition may favor an overproduction of energetic substrates to the fetus particularly when combined with abnormal sensing of the metabolic sufficiency by the placenta. Consequently, development of the fetus may be directed toward hyperinsulinism, increased fat accumulation (adipogenesis), disrupted regulation of appetite, reduced flexibility of metabolism. Crucially, the placental response to maternal obesity is distinctly driven by lipotoxicity and chronic low-grade inflammation rather than hyperglycemia. Unique molecular signatures of obesity-exposed placentas include the excessive accumulation of lipids within trophoblasts and the dysregulation of peroxisome proliferator-activated receptor gamma (PPARγ), a master regulator linking maternal adiposity to placental metabolic pathology (64). These condition-specific upstream events prioritize lipid storage over transport, thereby establishing a unique obesogenic intrauterine environment.

### Gestational diabetes mellitus and dysregulated glucose-lipid handling

Gestational Diabetes Mellitus (GDM) results in an entirely unique yet at times overlapping form of placental stress (65, 66). GDM’s impact on placental substrate utilization and hormonal activity stems from the combination of maternal hyperglycemia, altered lipid metabolism, insulin resistance, and increased inflammatory signaling (67). High levels of circulating glucose are present during GDM; however, these high glucose levels are accompanied by a larger number of alterations in overall maternal metabolism including disturbances in fatty acid flow and redox balance. These disturbances likely result in abnormal lipid uptake and processing by the placenta affecting fetal hepatic metabolism, fat cell formation and ultimately long term predisposition to obesity.

The placenta in pregnancies complicated by GDM will often demonstrate increased oxidative stress and inflammation, disrupted mitochondria function and decreased production of normal hormones (12, 68). All of these factors could increase the effect of IUGR/IGF caused by increased glucose transfer across the placenta. Insulin itself crosses the placenta poorly so therefore placental signaling mechanisms become critical in translating maternal glucose metabolism into fetal growth and metabolic adaptation (69). Abnormal placental function associated with GDM may facilitate excessive fetal growth; however this represents one aspect of the broader implications of placental dysfunction (70). Altered intrauterine glucose or lipid signaling, even without excessive birthweight, may alter the development of pancreas, appetite center, adipocytes and postnatal insulin sensitivity. In contrast to obesity, the primary placental insult in GDM is the adaptation to a hyperglycemic and glucolipotoxic milieu. Specific molecular hallmarks of GDM include the marked upregulation of facilitative glucose transporters and amino acid transporters to accommodate the excess maternal nutrients. Additionally, the diabetic environment promotes the stabilization of hypoxia-inducible factor 1-alpha (HIF-1α) and alters placental ceramide metabolism, representing distinct initiating triggers that differentiate GDM from hypertensive or obese pregnancies (71).

### Hypertensive disorders of pregnancy and uteroplacental malperfusion

The placenta is also affected by decreased blood flow and unstable blood flow to the placenta in hypertensive diseases (preeclampsia and chronic hypertension) through a mechanism of “vascular dysfunction” (72, 73). This vascular dysfunction causes trophoblastic cells to invade the uterus abnormally, it impairs normal spiral artery development (the arteries that supply the placenta) and results in a decrease in adequate blood flow to the placenta (74, 75). All these abnormalities cause an increase in oxidative stress, inflammation, damage to the syncytiotrophoblast layer, and lead to endothelial dysfunction. Thus, the placental environment created during hypertensive pregnancy has the ability to impact fetal growth due to either restricted delivery of oxygen and/or nutrient delivery at times or at irregular intervals. As such, the fetus may experience fluctuations in signaling related to stress (such as cortisol), glucocorticoids may be dysregulated, inflammatory responses may be activated, and the placenta’s priority for transporting substances may be altered (76).

Maternal stress could result in alterations to glucocorticoids metabolized by the placenta, reduced protection by enzymatic barriers and altered expression of genes responsible for trophoblast differentiation and endocrine secretion. Exposure to inflammatory signals can trigger cytokine production and recruitment of macrophages into the placenta, increase levels of oxidative stress and facilitate communication between the immune system and endocrine system of the placenta which ultimately affects nutrient transfer and blood vessel activity. As a consequence of these changes in the placenta, a fetus will experience different levels of stress hormones, inflammatory mediators, and regulatory signals related to growth (77). Therefore, maternal psychological stress and inflammation demonstrate that maternal neuroendocrine and immune homeostasis can provide additional means through which endocrine-metabolic risks can arise independent of obvious imbalances in nutrient intake or vascular disease. Unlike the nutrient-excess states of obesity and GDM, hypertensive disorders are fundamentally characterized by uteroplacental hypoperfusion and placental ischemia. The specific molecular drivers in this context involve the failure of extravillous trophoblasts to adequately remodel maternal spiral arteries, leading to a distinctive anti-angiogenic profile (78). This hypoxic and ischemic insult triggers unique adaptive and maladaptive responses that prioritize fetal survival over anabolic growth.

In summary, while maternal obesity, GDM, and hypertension originate from fundamentally distinct pathophysiological triggers—ranging from hyperlipidemia and hyperglycemia to placental hypoxia—they eventually converge on several shared downstream effector pathways. The terminal common denominators linking these diverse maternal complications include the induction of oxidative stress, endoplasmic reticulum (ER) stress, and mitochondrial dysfunction (79). Recognizing this distinction between the unique initiators and the shared executors is critical for developing targeted therapeutic interventions. It may be helpful to summarize the significant maternal disorders previously described before comparing them regarding their primary disruptions to the placenta as well as their probable endocrine-metabolic impacts on the offspring. It may be helpful to summarize the significant maternal disorders previously described before comparing them regarding their primary disruptions to the placenta as well as their probable endocrine-metabolic impacts on the offspring (**Table 2**).

**Table 2:** Comparative summary of maternal complications, principal placental alterations, and likely offspring metabolic outcomes.

Maternal complication	Principal placental alterations	Likely offspring metabolic outcomes
Maternal obesity (63)	Lipid accumulation, inflammatory activation, altered nutrient sensing, mitochondrial stress, dysregulated endocrine signaling	Increased adiposity, insulin resistance, altered appetite regulation, greater obesity risk
Gestational diabetes mellitus (70)	Enhanced glucose transfer, disturbed glucose-lipid handling, oxidative stress, inflammatory signaling, altered transporter expression	Fetal hyperinsulinemia, excess fat accretion, impaired glucose homeostasis, future type 2 diabetes susceptibility
Hypertensive disorders of pregnancy (68)	Uteroplacental malperfusion, hypoxia-related stress, antiangiogenic imbalance, endothelial dysfunction, altered transport efficiency	Growth restriction, catch-up growth vulnerability, insulin resistance, elevated cardiometabolic risk
Maternal stress and inflammation (58)	Cytokine activation, oxidative stress, altered glucocorticoid handling, immune-endocrine dysregulation	Altered stress-metabolic programming, adiposity risk, insulin sensitivity changes, appetite and neuroendocrine dysregulation
Endocrine disruption (37)	Abnormal steroid signaling, receptor perturbation, disrupted trophoblast endocrine function, altered gene regulation	Disturbed metabolic set points, altered adipogenesis, impaired hormonal regulation of energy balance

## Paternal metabolic programming: the intergenerational impact via placental epigenetics and function

While the preceding sections have extensively detailed the profound impact of maternal physiology on placental development, a growing body of evidence underscores that the paternal contribution extends far beyond the mere provision of haploid DNA. The paradigm of the Paternal Origins of Health and Disease (POHaD) recognizes that paternal metabolic health prior to conception is a critical determinant of placental phenotype and offspring disease risk (80, 81).

### Epigenetic vectors in spermatozoa

Experimental models robustly demonstrate that paternal obesity, type 2 diabetes, and exposure to high-fat diets induce significant epigenetic remodeling within spermatozoa (82, 83). These alterations encompass changes in DNA methylation patterns, histone modifications (particularly affecting H3K4me3 and H3K9me2), and the repertoire of small non-coding RNAs (such as microRNAs and tsRNAs). Crucially, these sperm-borne epigenetic factors are not inert; upon fertilization, they act as vectors that reprogram the embryonic epigenome and subsequently influence the transcriptional landscape of the developing placenta (82, 84).

### Placental phenotypic consequences

The transmission of these perturbed epigenetic signatures manifests distinctly within the placenta. Studies consistently report that the offspring of metabolically compromised fathers exhibit placentae characterized by increased hypoxia, endoplasmic reticulum (ER) stress, and impaired vascular development (reduced vascular density and abnormal labyrinthine structure) (85, 86). Furthermore, paternal metabolic stress has been shown to directly alter placental inflammatory signaling and the expression of nutrient transporters (such as SLC family genes), thereby compromising the placental capacity to regulate fetal growth and macro-nutrient delivery (84, 87).

### Sex-specific vulnerability and long-term offspring outcomes

Notably, these effects frequently display sex-specific vulnerability. Male placentae appear particularly susceptible to the adverse effects of paternal obesity, exhibiting more pronounced ER stress and inflammatory signaling compared to female counterparts (85, 88). The impact of paternal metabolic status is not limited to placental morphology; it correlates with systemic metabolic dysregulation in the offspring, including hepatic ER stress, altered gluconeogenic pathways, impaired glucose tolerance, and dyslipidemia (85, 89). Consequently, paternal metabolic programming predisposes the next generation to insulin resistance and obesity later in life, irrespective of maternal health or postnatal diet.

Although the majority of mechanistic insights currently derive from rodent models, emerging human epidemiological data support analogous associations, suggesting that paternal BMI and metabolic status prior to conception are correlated with altered placental efficiency and birth weight centiles (81). Therefore, integrating paternal metabolic programming into the conceptual framework of Developmental Origins of Health and Disease (DOHaD) is imperative. The placenta must be viewed not only as a translator of the maternal environment, but as the pivotal convergence point where both parental lineages shape the developmental trajectory of the offspring.

## Molecular pathways linking placental dysfunction to offspring endocrine-metabolic risk

### Placental nutrient sensing and transport pathways

Nutrient detection and transport by the placenta, are two of the earliest and most influential factors for fetal metabolic programming (90). These processes determine how many nutrients reach the fetus, and which nutrients will have priority based on a number of different physiological states (e.g., hormonal or metabolic) under which the nutrient is delivered to the fetus. The main mechanism used by the placenta to sense maternal energy reserve levels includes signaling pathways like Mechanistic Target of Rapamycin (mTOR) and adenosine monophosphate-activated protein kinase (AMPK) (91). Both mTOR and AMPK can provide an integrated picture of available nutrients, cellular energy stores, insulin mediated signaling, growth factors present, and stress.

In both maternal obesity and GDM (gestational diabetes mellitus) there is a frequent shift in nutrient sensing pathways so that they perceive a surplus of nutrients; which promotes a greater transfer of glucose, amino acids and fats from mother to fetus (70). Frequently this occurs through an increase in the amount of glucose transporters, amino acid transport systems, proteins responsible for fatty acid uptake into cells and those involved with intracellular fat handling. Often the result of these changes is not merely more substrate available to the fetus but an alteration in the fetal endocrine environment characterized by increased levels of fetal insulin, increased rate of lipid synthesis and accelerated accumulation of fat (42). Both of these factors tend to encourage the fetus to store more fat than usual, place additional burden on the fetal pancreas’ beta-cells and decrease the metabolic flexibility of the fetus (92).

### Inflammatory signaling and immune activation

Placental inflammatory response typically occurs by way of pathways including NFκB (nuclear factor kappa B), JNK (c-Jun N-terminal kinase) pathway activation, inflammasome activation and cytokines such as TNFα (tumor necrosis factor-alpha), IL-6 (interleukin-6) and IL-1β (interleukin-1beta) and other associated mediator networks (93). Activation of these pathways in the context of an obese pregnant woman’s state can lead to disruption of insulin signaling, decreased trophoblast function and impaired mitochondrial function and alteration of regulatory mechanisms of nutrient transporters. Obesity-related chronic low-level systemic inflammation combined with excess lipid could result in a metabolically overburdened placenta that is also immune-stimulated, thus potentially increasing fetal exposure to both a proadipogenic and IR (insulin resistant) environment (69).

The activation of immune responses may also have developmental effects on neuroendocrine functions. The release of inflammatory mediators from the placenta during pregnancy can modify the hypothalamus’s signaling for food intake, modify the fetal stress response, and modulate the differentiation of fat cells (94). These mechanisms may contribute to long-term obesity, abnormal feeding behaviors, reduced insulin sensitivity, and other metabolic disorders. Inflammation can cause lasting transcriptional alterations in the placenta as well, thus increasing the time period that maternal exposure is acting on (95). Therefore, instead of just being a sign of disease in mother, placental inflammation will be a mechanism causing long-lasting reorganization of fetal endocrine-metabolic system function.

### Endoplasmic reticulum stress

The presence of ER stress adds to this as well because it disrupts secretory activity, protein folding, and stress signaling (96). The accumulation of unfolded/misfolded proteins activates the Unfolded Protein Response (UPR) in an attempt to restore cellular homeostasis (97). However, persistent UPR activation can result in suppression of protein synthesis, altered endocrine output, impaired trophoblast differentiation, and interactions with inflammatory and oxidative pathways resulting in increased placental dysfunction (98). Specifically, these conditions are exacerbated under conditions of nutrient excess, inflammation, or hypoxia. Collectively, oxidative stress, mitochondrial-impaired function, and endoplasmic-reticulum stress contribute to creating a placenta where there has been significant alteration to the quality of fetal exposure.

### Angiogenic imbalance, hypoxia signaling, and vascular remodeling

Placental vascular development is central to fetal metabolic programming because adequate perfusion ensures reliable delivery of oxygen and nutrients during gestation (99). This depends on coordinated trophoblastic invasion, spiral artery remodeling, endothelial adaptation, villous angiogenesis, and vascular branching (100). When these processes are disrupted, as in preeclampsia, chronic hypertension, or placental malperfusion, the consequences extend beyond reduced blood flow. Abnormal placental vascular function also promotes angiogenic imbalance, endothelial dysfunction, oxidative stress, activation of hypoxia-responsive pathways, and structural changes in the exchange surface (101). Together, these changes alter both the quantity and biological meaning of fetal exposure to oxygen, nutrients, and signaling molecules, thereby shaping long-term endocrine and metabolic outcomes.

Angiogenic imbalance, particularly a shift toward anti-angiogenic signaling and reduced vascular plasticity, may impair villous development, lower capillary density, and increase nutrient diffusion distance (72). Intermittent placental hypoxia then activates hypoxia-inducible pathways that reprogram placental metabolism, inflammation, redox balance, and transport (102). Although some adaptations may support immediate fetal survival, they may do so at the expense of optimal metabolic maturation. Sustained impairment of oxygen delivery can adversely affect development of the fetal pancreas, liver, muscle, and vasculature, increasing later risk of insulin resistance and cardiometabolic disease (103). Vascular dysfunction also interacts with oxidative stress, inflammation, hormone production, and cortisol metabolism, making it a central integrative mechanism through which placental dysfunction programs long-term disease risk, even when birth weight is not markedly reduced.

### Epigenetic remodeling, non-coding RNAs, and transcriptional memory

The placenta undergoes extensive epigenetic remodeling throughout gestation, making it highly responsive to maternal metabolic, nutritional, inflammatory, and hormonal conditions (7). MicroRNAs and other non-coding RNAs are now recognized as key regulators of placental plasticity. They influence invasion, syncytiotrophoblast formation, inflammation, mitochondrial function, transport, and hormone production, and some are released in exosomes that may signal to maternal tissues (104). These molecules may therefore function both as mechanistic mediators and potential biomarkers of placental dysfunction. This systematic view shown in **Figure 2** will be key to understanding how clinically distinct maternal complications can produce developmental outcomes that have some elements of overlap between offspring (105).

**Figure 2:**
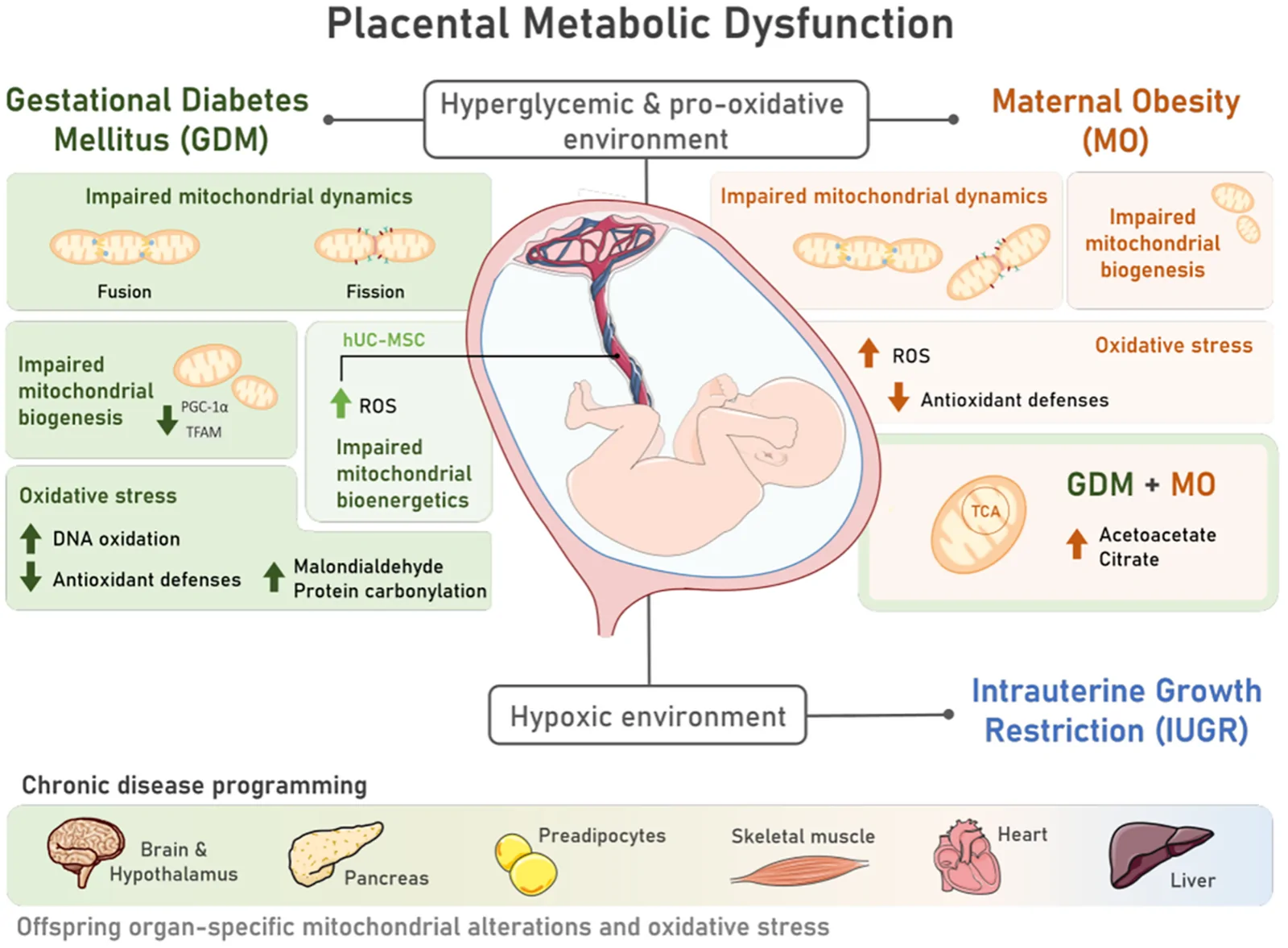
Placental metabolic dysfunction in gestational diabetes mellitus and maternal obesity: mitochondrial dynamics, biogenesis, oxidative stress, and downstream chronic disease programming.

Placental metabolic dysfunction during pregnancies characterized by the presence of Gestational Diabetes Mellitus (GDM) and Maternal Obesity (MO) includes mitochondrial structural and functional alterations and oxidative stress, contributing to offspring chronic disease programming. The characteristic hyperglycemic and pro-oxidative intrauterine environment of pregnancies complicated by GDM and MO induces placental metabolic dysfunction via alterations in mitochondrial dynamics, with unbalanced fission and fusion events; mitochondrial biogenesis, via decreased peroxisome proliferator-activated receptor gamma coactivator 1-alpha (PGC-1α) and mitochondrial transcription factor A (TFAM) levels; and impaired mitochondrial bioenergetics, which was also observed in multipotent human umbilical cord mesenchymal stem cells (hUC-MSC) of GDM pregnancies. Increased reactive oxygen species (ROS) are also common to both placenta and hUC-MSC tissues in both pregnancy disorders. Placental oxidative stress is further marked by reduced antioxidant defenses and increased DNA oxidation, protein carbonylation, and malondialdehyde levels in GDM placentas. MO during a GDM pregnancy may further contribute to mitochondrial dysfunction, as increased levels of metabolites involved in the tricarboxylic acid (TCA) cycle and ketogenesis (citrate and acetoacetate, respectively) were detected. This compromised intrauterine environment can progress into a hypoxic state, increasing the risk of Intrauterine Growth Restriction (IUGR) development. GDM, MO, and IUGR have been associated with an increased risk of offspring chronic disease. Mitochondrial alterations and oxidative stress, which are intimately involved in chronic diseases, were observed in human and animal studies in different tissues of GDM and MO offspring, such as the brain and hypothalamus, pancreas, preadipocytes, skeletal muscle, heart, and liver, highlighting the role of metabolic pregnancy disorders in disease programming (105).

### Structured synthesis of placental programming

Moving beyond descriptive pathology, a structured synthesis reveals three convergent dimensions across maternal complications. First, nutrient-sensing flux exhibits quantitative distinctions: GDM upregulates GLUT1 and amino acid transporters, conferring a 2- to 4-fold increased risk of offspring T2DM (106). Maternal obesity triggers placental lipotoxicity, where each 5-unit increase in pre-pregnancy BMI raises offspring obesity odds by 1.12- to 1.20-fold (107). Second, the epigenetic dimension provides stable memory; placental epigenetic clocks correlate with elevated systolic BP in childhood. Third, mitochondrial dysfunction (reduced PGC-1α/TFAM) acts as a central integrator, shifting placental priority from anabolic support to cellular survival across diverse pathologies.

## Fetal and offspring consequences of placental programming

It is imperative to distinguish placental programming from simple alterations in fetal size. While placental dysfunction frequently results in growth restriction or overgrowth, its primary impact is the deterioration of metabolic qualityand endocrine regulation. The placenta acts as a conductor of fetal organ maturation, fine-tuning pancreatic insulin secretion, adipocyte differentiation, and hepatic metabolism. Therefore, offspring may exhibit a normal birth weight while harboring profound deficits in metabolic flexibility and stress responses. This functional programming, rather than growth per se, constitutes the core mechanism driving intergenerational metabolic risk.

Placental programming has major implications for fetal development and long-term metabolic health. The intrauterine environment is determined by the placenta during a period of high developmental plasticity; therefore, disruptions to placental function will alter the balance between nutrients, oxygen, hormones, cytokines, stress-related signals etc., reaching the fetus (108). This will have an impact on not only the growth of the fetus but also the maturation of key endocrine and metabolic tissues including the pancreas, adipose tissue, liver, skeletal muscle, and neuroendocrine systems involved in appetite and energy balance (61). Consequently, offspring may be born with a latent susceptibility to obesity, dysglycemia, insulin resistance, dyslipidemia and broader cardiometabolic disease. It is important to recognize that diverse conditions experienced by mothers (obesity, gestational diabetes, placental insufficiency, undernutrition, hypertensive disease) can converge on overlapping pathways in the placenta and produce similar phenotypes in their offspring.

### Programming of pancreatic development and glucose homeostasis

The fetal pancreas has very high sensitivity to the changes in placental function as the stability of the intrauterine environment significantly influences the mass of beta-cells, islet structure and glucose sensing capability (109). Dysfunction of the placenta produces changes in the levels of glucose, lipid, glucocorticoid and inflammatory mediator that are transferred from mother to fetus; these changes affect both the process of differentiation of the pancreas and the maturation of its endocrine function (110). Maternal obesity and gestational diabetes lead to increased transference by the placenta of glucose and lipid to the fetus which can result in prolonged hyperglycemia and hyperinsulinism in the fetus (55). Hyperinsulinism produced in response to initial hyperglycemia can provide for normal growth of the fetus. However prolonged stimulation of beta cells during development may alter glucose homeostasis and therefore create greater susceptibility to impaired glucose tolerance (IGT) and type 2 diabetes (111). Conversely placental insufficiency, maternal malnutrition, and hypertensive disease can limit substrate delivery to the fetus while increasing fetal stress hormone levels (112). These conditions may prevent adequate vascularization of islets in addition to limiting insulin secretory capacity. Regardless if the fetus experiences hyper- or hypo-nutrient status, adaptations made to an abnormal intrauterine environment will likely decrease the flexibility of the endocrine system and contribute to future metabolic dysfunction.

### Adipose tissue development, appetite regulation, and obesity susceptibility

Fetal adipose programming has been identified as a significant consequence of impaired placentation (113). Programming of fetal fat development is highly dependent upon gestational nutrient and hormone signals. The fetus develops into a state of increased propensity for adipocyte formation, lipid deposition and decreased energy expenditure when placental transport results in an excess of glucose and fatty acids along with abnormal levels of inflammatory mediators and adipokines (114). Increased adiposity at birth does not represent the primary impact; rather, it is the establishment of the trajectory for developing future obesity and decreased metabolic flexibility that will have a greater long-term effect.

Placental dysfunction also impacts the degree to which the fetus is exposed to various signals including leptin, glucocorticoids, insulin related signals and inflammation which all contribute to the formation of the hypothalamus’s regulatory systems controlling satiation, reward, and energy expenditure (115). Therefore, offspring may develop an enhanced body fat accumulation as well as an imbalance in their food intake (increased preference for high caloric foods) and decreased ability to recognize fullness. This can account for why there is an increased risk of developing obesity even if birth weight is relatively normal.

### Hepatic lipid metabolism and dyslipidemic risk

The fetal liver is an important target for placental programming due to its central role in regulating lipid biosynthesis, glycogen stores, oxidative metabolism and endocrine-metabolic functions during early development (116). Changes in placental transport of nutrients will determine the type and amount of substrates available to the fetal liver which in turn can shape its long term metabolic set point (117). As such, in the case of pregnancy complicated by maternal obesity or gestational diabetes, enhanced placental transport of glucose and free fatty acid may lead to an overexposure of the fetal liver to nutrients resulting in lipogenesis, aberrant insulin signalling, hepatomegaly/hepatic fat deposition and eventually dyslipidemia (118). On the other hand, fetal growth restriction and undernutrition cause programming of hepatic metabolism, however this occurs through scarcity (i.e., reduction) as opposed to excess (119).

### Sex-specific responses and life-course variability

The effects of placental programming on offspring can vary depending upon sex due to sexually dimorphic responses of placentas. This could have implications for how well an individual adapts to maternal complications. Therefore, male and female fetuses that are exposed to the same maternal complication would have different metabolic adaptations to that insult (24). Phenotypes created via placental programming will vary throughout the lifespan. For example, some phenotypes may appear immediately, i.e., changes in fat mass (adiposity) or levels of insulin in cord blood. Other phenotypes may take longer to become apparent through interactions with dietary patterns, pubertal development, growth patterns and/or lifestyles. In other words, placental programming provides a biological predisposition for an outcome; it does not provide a definitive destiny (120). Therefore, a fetus that is programmed for “metabolic thrift” may remain clinically normal until placed in an environment rich in calories. Similarly, a fetus that is exposed to excessive amounts of calories during gestation may eventually develop obesity and insulin resistance as they age. Ultimately, the long term phenotype of an individual will depend on both the prenatal placental programming and their postnatal environment Before moving to translational and clinical implications, **Figure 3** visualizes how placental programming affects multiple fetal organs simultaneously (121).

**Figure 3:**
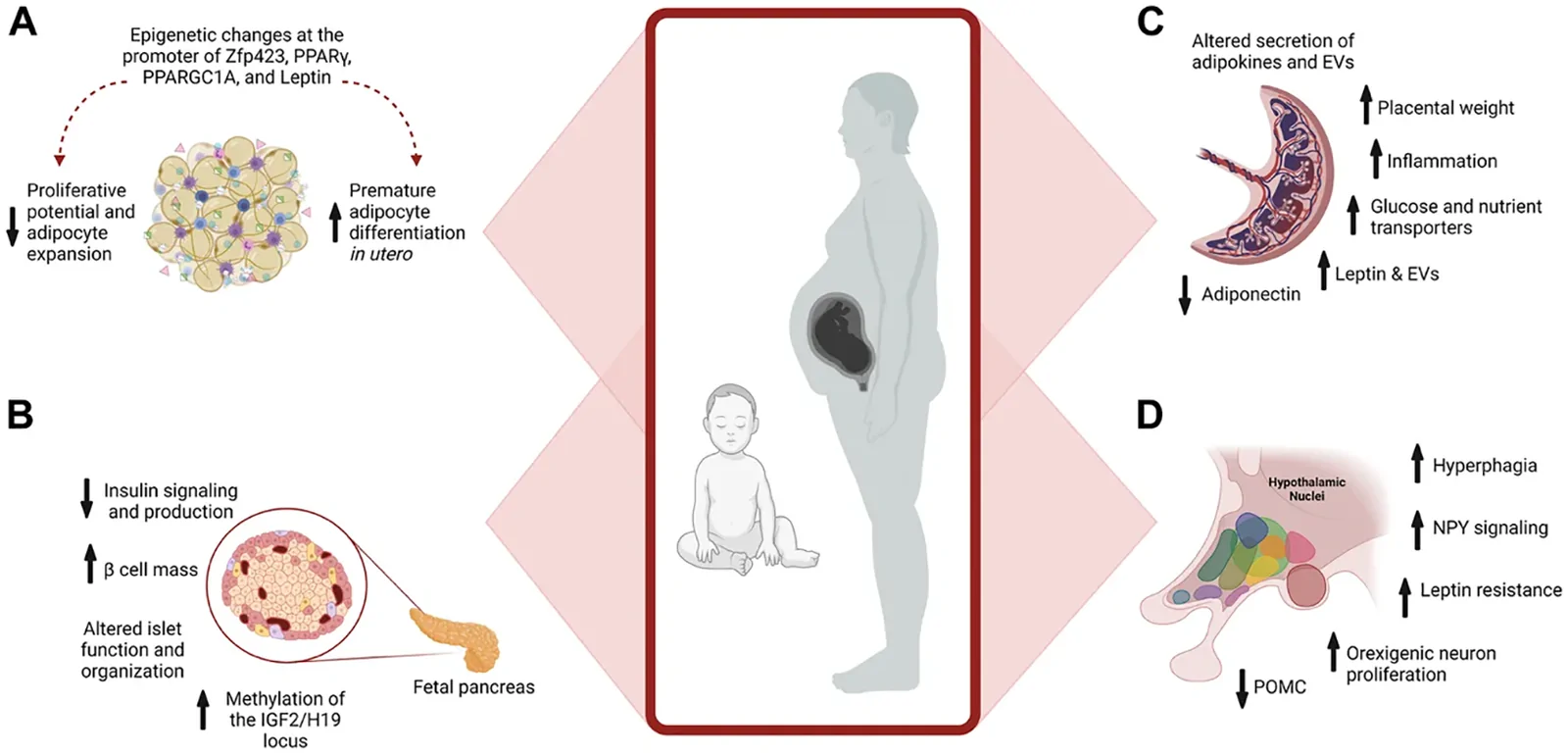
Effects of maternal obesity on fetal development. **(A)** Epigenetic changes in adipose tissue affecting proliferation and differentiation; **(B)** fetal pancreas changes with altered insulin signaling, cell mass and function; **(C)** altered placental secretions increasing inflammation and nutrient transport but decreasing adiponectin; **(D)** hypothalamic nuclei changes with increased appetite signaling and leptin resistance.

## Translational and clinical perspectives

Translating placental programming into clinical practice requires moving beyond simple associations between maternal complications and later offspring metabolic disease. Maternal diagnoses such as obesity, gestational diabetes, hypertensive disorders, and abnormal fetal growth remain clinically useful, but they do not capture the underlying biology of placental dysfunction. Similar maternal phenotypes may reflect very different placental states, while distinct pregnancy complications may converge on shared mechanisms such as inflammation, oxidative stress, vascular maladaptation, endocrine disruption, and epigenetic remodeling. For this reason, future progress depends on pathway-based risk stratification that identifies biologically meaningful placental dysfunction during pregnancy and links it to later endocrine-metabolic risk in the child.

A key translational goal is the development of antenatal placental biomarkers for early risk stratification. Candidate biomarkers include placental proteins, angiogenic and inflammatory mediators, extracellular vesicles, circulating nucleic acids, microRNAs, epigenetic signatures, and maternal metabolic profiles that reflect placental activity. Their clinical value lies in providing indirect evidence of trophoblast stress, vascular imbalance, endocrine disturbance, and altered transport before permanent fetal programming occurs. However, because placental dysfunction arises from interacting biological networks rather than isolated pathways, multi-biomarker panels will likely outperform single markers. Major barriers include lack of assay standardization, uncertainty regarding optimal gestational timing, and insufficient validation across large and diverse populations.

Concrete biomarkers are moving beyond maternal diagnosis. Cord blood metabolomics​ identifies specific lipid metabolites that predict childhood obesity risk (122). Placental epigenetic signatures also show high specificity for identifying at-risk infants. However, sampling heterogeneity poses a major barrier; spatial compartmentalization of the placenta obscures true signals without standardized protocols. Confounding by indication further complicates isolation of metabolic stress effects. Clinically, these findings mandate a shift to pre-conceptional counseling. Optimizing maternal BMI and glucose beforepregnancy remains the most feasible, high-impact intervention to mitigate lifelong metabolic trajectories.

Human longitudinal cohort studies remain essential because they connect maternal phenotype, placental biology, and child follow-up within the same developmental context. Yet these studies are often limited by short follow-up periods, inconsistent placental sampling, inadequate control of postnatal influences, and insufficient power for sex-specific analyses. Multi-omics approaches, including transcriptomics, epigenomics, proteomics, metabolomics, and lipidomics, offer higher-resolution characterization of placental heterogeneity. At the same time, experimental systems such as animal models, trophoblast organoids, ex vivo placental perfusion, and placenta-on-chip platforms are needed to test causality and evaluate therapies. The strongest future research will integrate cohort-based discovery with experimental validation.

Preventive strategies are most effective before placental dysfunction is established. Preconception care and early pregnancy optimization of maternal nutrition, weight, physical activity, glucose control, and vascular health may reduce placental inflammation, abnormal nutrient transfer, and endocrine imbalance. Looking ahead, therapies may need to target the dominant biological pathway involved, whether vascular, inflammatory, glucocorticoid-related, or nutrient-sensing. Importantly, intervention studies should move beyond short-term obstetric outcomes and include placental biomarkers together with long-term offspring metabolic follow-up.

## Future directions

Future studies examining the placental programming of metabolic diseases will need to evolve past general correlations and provide a precise understanding of mechanisms. One important area of focus is developing cohorts of mothers-infants placentas-children that include detailed maternal characteristics, as well as standardized samples of placenta in addition to long term follow up of children through their later childhood/adolescence. There are several reasons this is necessary; many programmed metabolic processes do not have clinical manifestations at birth; therefore, assessing these processes through short-term neonatal outcomes does not adequately represent the later risk for insulin resistance, obesity susceptibility, abnormal lipid profiles, or aberrant endocrine functions. Future cohort study design must also allow for the inclusion of postnatal factors such as infant nutrition and feeding patterns, infant growth rates, dietary intake, levels of physical activity, and social/economic status exposure to determine if there were specific prenatal placental effects. Current translational barriers and priority directions are summarized in **Table 3**.

**Table 3:** Research gaps, translational barriers, and priority directions.

Domain	Current translational barrier	Priority direction
Biomarker discovery	Many candidate markers are studied in small or single-center cohorts and lack reproducibility	Multi-center validation with standardized sampling windows and assay harmonization
Placenta-child linkage	Most studies predict pregnancy complications rather than long-term offspring metabolic outcomes	Longitudinal cohorts connecting antenatal biomarkers, placental tissue profiles, and child endocrine-metabolic follow-up
Placental sampling	Tissue heterogeneity and inconsistent biopsy protocols reduce comparability across studies	Standardized spatially informed placental sampling and reporting frameworks
Molecular resolution	Bulk analyses obscure cell-type-specific dysfunction	Integration of single-cell, spatial transcriptomic, proteomic, and metabolomic approaches
Experimental modeling	No single model captures endocrine, immune, vascular, and transport complexity simultaneously	Complementary use of organoids, placenta-on-chip systems, animal models, and human cohort data
Intervention science	Maternal treatment trials often assess short-term obstetric outcomes only	Trials incorporating placental biomarkers and longer offspring metabolic endpoints
Clinical implementation	Maternal diagnosis remains a crude proxy for placental biology	Pathway-based risk stratification using combined maternal, placental, and fetal markers

A second focus is on improving placental characterization at the molecular and spatial scales. Much bulk placental analysis hides the fact that the placenta is an organ of heterogeneity with regionalized transport, hormone secretion, immune function, and vascular activity. The future use of single-cell RNA sequencing (transcriptomics), spatial RNA sequencing (spatial transcriptomics), proteomics, metabolomics and epigenetic sequencing will allow researchers to specify which cell populations and signaling networks are responsible for fetal development in response to maternal conditions. It will be critical to distinguish those commonalities across maternal complication states from condition-unique and sexually dimorphic pathways. Sexual dimorphism will need increased emphasis as well because male and female placentas frequently have different adaptations and can convey risk via different processes. Experimental modeling of the placenta needs to increase its integration into translational science. Animal models are still needed for causality, however, these models need to be supplemented with human trophoblast organoids, placenta-on-a-chip devices, and ex vivo placental perfused models which can provide a closer approximation to human placental function. A greater amount of research is expected to occur when there is alignment between human cohorts and the experimental data, thereby enabling direct testing of biomarker candidates and potential pathways as opposed to indirect inference. Biomarker identification/translation and clinical application are another area that has tremendous opportunity for future advancement. Biomarkers (multiple) based upon placental proteins, EVs, miRNA, DNA methylation signatures, and maternal metabolomics for risk assessment in early gestation have been identified. Future research efforts need to focus on validating biomarker sets within multiple diverse populations and linking biomarkers to both maternal obstetric complications and long-term metabolic health of offspring.

Ultimately, intervention research needs to move away from simply optimizing current pregnancy outcomes (obstetrics) and instead test if mother’s interventions will modify placental development/programming directly. Interventions could include preconceptional metabolic optimization, nutritionally based interventions, blood sugar management, reducing inflammation and using specific therapeutic pathways for each condition. All interventions need to be tested both by assessing placental function as well as the developing fetus over time. Ultimately, we would like to develop an integrated framework where maternal risks, fetal/placental biological factors and ongoing assessment of the newborn/fetus are used to prevent the inter-generational transmission of metabolic diseases.

## Conclusion

Placental programming provides a biological explanation for why maternal complications are translated from biologic events (maternal) into long-term endocrine-metabolic risks in offspring. Regardless of whether these maternal complications include obesity, gestational diabetes mellitus, hypertensive disorders of pregnancy, undernutrition, inflammation or endocrine stress, the placenta is not simply a passive conduit that allows for the transfer of nutrients across the uterus; rather it is a highly regulated organ capable of interpreting maternal signals and converting those maternal signals into developmental responses. The placenta accomplishes this by altering nutrient uptake, vascular exchange, endocrine function, immune response, oxidative balance and epigenetic regulation. These changes affect the maturation of pancreatic function, fat cells, liver function, skeletal muscle, and neuroendocrine systems, thereby establishing the foundation for the offspring’s propensity to develop dysglycemia, insulin resistance, obesity and dyslipidemia with subsequent cardiometabolic disease. A key understanding from this study was that many of the various maternal health issues share similar pathways through which they affect the placenta. This explains why there are so many variations of pregnancy that can result in very similar effects on the children born into those pregnancies; further it explains why being a certain size at birth does not fully capture how much developmental-metabolic risk has been established.

The impact of placental dysfunction is not just about how big or small a baby will be, but about the way that placental function affects the type of endocrine and metabolic system that develops for the fetus. While placental adaptations may support fetal survival and early postnatal growth, they may simultaneously predispose the infant to later vulnerability when the predicted postnatal environment differs from the actual environment encountered after birth.

The present evidence clearly demonstrates that the placenta represents a critical mechanistic site to explain the etiology of many metabolic diseases due to their developmental origin. There are still significant barriers to make greater strides in this area. These include the implementation of standardization of placental phenotypes; combining multi-omic analyses with prospective longitudinal human studies (which would provide valuable information about changes throughout gestation); developing better animal models to study placental function; and identifying specific placental biomarkers to assess if there is dysfunctional programming occurring early in pregnancy versus later post-delivery. Also important will be the initiation of clinical trials aimed at testing whether interventions designed to improve maternal-fetal-placental health will have an impact on both the short-term perinatal outcome, and the long-term metabolic destiny of offspring.
